# MSC-derived small extracellular vesicles overexpressing miR-20a promoted the osteointegration of porous titanium alloy by enhancing osteogenesis via targeting BAMBI

**DOI:** 10.1186/s13287-021-02303-y

**Published:** 2021-06-16

**Authors:** Wei Liu, Jinghuan Huang, Feng Chen, Dong Xie, Longqing Wang, Cheng Ye, Qi Zhu, Xiang Li, Xiaolin Li, Lili Yang

**Affiliations:** 1 Spine Center, Department of Orthopaedics, Shanghai Changzheng Hospital, Naval Medical University (Second Military Medical University), Shanghai, 200003 China; 2 Department of Orthopedic Surgery, Shanghai Jiao Tong University Affiliated Sixth People’s Hospital, Shanghai, 200233 China; 3 College of Chemistry and Chemical Engineering, Shanghai Jiao Tong University, Shanghai, 200240 China; 4 School of Mechanical Engineering, Shanghai Jiao Tong University, State Key Laboratory of Mechanical System and Vibration, Shanghai, 200240 China

**Keywords:** Mir-20a, Small extracellular vesicles, Overexpressing, Osteogenesis, Osteointegration, Synergetic effect

## Abstract

**Background:**

Patients with osteoporosis have a high risk of implant loosening due to poor osteointegration, possibly leading to implant failure, implant revision, and refracture. RNA interference therapy is an emerging epigenetic treatment, and we found that miR-20a could enhance osteogenesis. Moreover, small extracellular vesicles (sEVs) derived from bone marrow mesenchymal stem cells (hBM-MSCs) were utilized as nanoscale carriers for the protection and transportation of miR-20a (sEV-20a). In this study, we intended to determine whether sEVs overexpressing miR-20a could exert a superior effect on osteoporotic bone defects and the underlying mechanism.

**Methods:**

For evaluating the effect of sEV-20a on osteogenesis, in vitro and in vivo studies were performed. In vitro, we first showed that miR-20a was upregulated in the osteogenic process and overexpressed sEVs with miR-20a by the transfection method. Then, the proliferation, migration, and osteogenic differentiation abilities of hBM-MSCs treated with sEV-20a were detected by CCK-8 assays, alkaline phosphatase staining and alizarin red staining, qRT-PCR, and western blot. In vivo, we established an osteoporotic bone defect model and evaluated the effect of sEV-20a on bone formation by micro-CT, sequential fluorescent labeling, and histological analysis. To further explore the mechanism, we applied a bioinformatics method to identify the potential target of miR-20a.

**Results:**

In vitro, sEV-20a was successfully established and proved to promote the migration and osteogenesis of hBM-MSCs. In vivo, sEV-20a promoted osteointegration in an osteoporotic rat model. To further elucidate the related mechanism, we proved that miR-20a could enhance osteogenesis by targeting BAMBI.

**Conclusions:**

Collectively, the in vitro and in vivo results confirmed that MSC-derived sEV-20a therapy effectively promoted osteoporotic porous titanium alloy osteointegration via pro-osteogenic effects by targeting BAMBI.

**Supplementary Information:**

The online version contains supplementary material available at 10.1186/s13287-021-02303-y.

## Background

Osteoporosis, which often occurs in spine, distal of the wrist and hip, is a disease featured by compromised bone strength and increased risk of fractures and contributes to a heavy economic burden to families and societies [1]. Internal fixations or joint replacements have been applied to improve the survival rate and quality of life of patients with fragile bone fracture. However, the efficacy of internal fixations or joint replacements is compromised in osteoporotic patients due to implant loosening caused by poor osteointegration [2]. Implant loosening could result in many adverse consequences, such as implant failure, which are detrimental to the recovery of patients [3]. A study demonstrated that 60% implant failure could be found in osteoporotic patients [4]. Therefore, strategies are urgently needed to enhance osteointegration and prevent implants from loosening.

Ideal osteointegration requires a proper balance between osteogenesis and osteoclastogenesis, which enhances osteogenesis or reduces osteoclastogenesis. In this study, we proposed a combination of biological and engineering approaches to enhance osteogenesis. RNA interference (RNAi) therapy is a newly developed epigenetic treatment that utilizes RNA as a kind of drug to precisely target key genes and is based on the negative regulatory principles that downregulate the expression of antagonists to influence some biological processes [5, 6]. MicroRNAs (miRNAs) are well conserved, endogenously synthesized RNA molecules for RNAi therapy that could target mRNA for the inhibition of transcription or RNA cleavage [7]. Synergistic RNAi therapy was applied to improve local bone quality, and RNA could also be incorporated into biocompatible scaffolds for tissue regeneration applications [8, 9]. However, no study has been carried out for exploring the effects of miR-20a on osteointegration for osteoporosis until now. Thus, in our research, we aimed to prove that miR-20a-5p (miR-20a) could be an appropriate RNAi therapy molecule for osteointegration.

Nevertheless, miRNAs are unstable and easily degraded by serum enzymes before entering target cells. Hence, we need an effective nanoscale carrier for the transportation of miR-20a. Small extracellular vesicles (sEVs), nanoscale extracellular vesicles secreted by most cells, are composed of a lipid bilayer that encloses various internal cargos such as RNA, DNA, and proteins, mediating intercellular communication in both physiological and pathological processes [10–12]. Based on the internal cargos, sEVs could be functionally improved via modification of the internal nucleic acids or proteins through cell engineering, which is termed “modularized sEVs,” making sEVs ideal carriers of RNAi therapy molecules [13]. Therefore, we fabricated hBM-MSC (human bone marrow mesenchymal stem cell)-derived sEVs overexpressing miR-20a (sEV-20a) for miRNA delivery.

Porous titanium alloys with a lower elasticity modulus and much greater surface area than other materials have been applied as engineering scaffolds to improve their fit in osteoporotic conditions [14, 15]. Hydrogels have widespread applications as therapeutic biological scaffolds due to their outstanding biocompatibility accompanied by sustained-release properties. More importantly, hydrogels could augment the retention and stability of sEVs, protecting sEVs from untimely degradation [16–21].

In this study, we fabricated engineered sEVs derived from hBM-MSCs overexpressing miR-20a to explore its role in osteogenesis and its potential therapeutic effects on osteoporosis. Our research results suggested that our strategy was efficient in promoting implant osteointegration by promoting osteogenesis.

## Material and methods

### MiRNA transfection

We acquired the miR-20a sequence by miRBase (http://www.mirbase.org/). hBM-MSCs overexpressing miR-20a were obtained by transfection method. Moreover, we applied a random sequence as a negative control (NC). hBM-MSCs were seeded in each well of 24-well plates. Then, the hBM-MSCs were transfected with miR-20a mimics and inhibitor by lipofectamine 3000 (Invitrogen). The culture medium was changed after 6 h to fresh complete culture medium, while the cells were harvested 48 h later after transfection.

### Isolation and identification of sEVs

#### sEV isolation

Ultracentrifugation was utilized to isolate sEVs of hBM-MSCs, including sEVs and sEV-20a. In short, when the cell confluence reached 80%, the cell supernatant was removed and replaced by serum-free medium. The medium was collected after incubation under different conditions 48 h later. The dead cells were removed by centrifugation at 300*g* and 2000*g* for 10 min and 15 min, respectively. After that, the supernatant was filtered through a filter with a diameter of 0.22 μm (Micropore). Then, the filtered supernatant was centrifuged at 100,000*g* for approximately 1.5 h twice in Ultra-Clear™ tubes (Beckman Coulter, USA). Finally, after the abovementioned operation, we harvested the required pellets, which were resuspended in PBS, and the pellets were then kept at  -80 °C for further experiments.

#### sEV identification

Transmission electron microscopy (TEM, JEM-1400) was applied for the observation of the morphology of sEVs. Additionally, we detected the size distribution and the concentration by nanoparticle tracking analysis (NTA, ZetaView PMX 110, Particle Metrix). The concentration of sEVs was approximately 1×10^9^ -1×10^12^/ml. Western blotting to examine the specific surface markers TSG101 (14497-1-AP), CD9 (ab92726), and Alix (12422-1-AP) was applied for the identification of sEVs. Calnexin (ab133615) was used as a negative marker, and GAPDH (ab8245) was applied as an internal reference.

### In vitro responses to sEV-20a

#### Cell culture

In this study, we obtained hBM-MSCs from the cell bank of SIBS (Shanghai, China). hBM-MSCs were incubated with complete α-MEM (Gibco, 10% fetal bovine serum (FBS, Gibco)). hBM-MSCs were incubated in a humid atmosphere (37 °C, 5% CO_2_).

#### Cell proliferation and migration

Three groups were established for the experiments: the Control group, sEV group, and sEV-20a group. The number of sEVs in the sEV group and sEV-20a group was the same, and sEVs and sEV-20a were added in complete culture medium in an equivalent amount to that of the control group. The proliferation of hBM-MSCs was detected by CCK-8 assays. In summary, the two kinds of cells were seeded in a well of a 96-well plate at an initial density of approximately 2 × 10^3^ cells/well. After treated with PBS, sEVs, and sEV-20a for 1, 3, and 7 days, we removed the culture medium and replaced it with fresh culture medium and 10% CCK-8 solution (Cell Counting Kit-8). After incubation for 2 h at 37 °C, the absorbance value was assessed by using a microplate reader (Epoch, Bio-Tek, USA) at 450 nm.

Transwell assays were used for evaluating cell migration. hBM-MSCs (2 × 10^4^) were loaded into the upper chamber, while culture medium, sEVs, and sEV-20a were added to the lower chamber in a Transwell plate (Millipore). Twenty-four hours later, we removed the cells in the upper chamber with a cotton swab and treated the lower chamber with 4% paraformaldehyde for fixation for approximately 20 min. Then, 0.5% crystal violet was applied to stain the migrated cells for approximately 5 min. The migrated cells were photographed by light microscopy (Olympus IX 70, Tokyo, Japan).

#### Osteogenic differentiation

ALP staining, alizarin red staining (ARS), and mRNA relative levels of ALP, runt-related transcription factor 2 (RUNX2), and osteocalcin (OCN) were utilized for the assessment of osteogenic differentiation. We applied osteogenic induction medium (OM) supplemented with PBS, sEVs, and sEV-20a to induce hBM-MSCs to conduct ALP and ARS, respectively. At each time point, the cells induced by OM were treated with 4% paraformaldehyde for fixation. ALP or ARS solution was applied for approximately 10 min. After the samples were washed with ddH_2_O, they were recorded via light microscopy (Olympus IX 70, Tokyo, Japan). qRT-PCR was applied to determine the relative mRNA levels of ALP, OCN, and RUNX-2. Generally, TRIzol reagent (Invitrogen) was utilized to obtain total RNA from cells induced by OM. Subsequently, complementary DNA (cDNA) was obtained by reverse transcription from total RNA using the PrimeScript RT reagent Kit (Takara). Subsequently, we applied SYBR Green detection reagent (Takara) was applied for further analysis. In the end, all the results were normalized to GAPDH, and 2^^-△△CT^ was used for the calculation of the relative expression levels of mRNA.

### Fabrication of porous titanium alloy

Samples were fabricated using a selective laser melting (SLM) machine (M2, Concept Laser, Germany). The Ti_6_AL_4_V powder (grade 5, Concept Laser, Germany) was applied for SLM process. The chemical composition of the power is 88.35–90.60 wt.% for Ti, 5.50–6.75 wt.% for Al, 3.50–4.50 wt.% for V, and 0.40 wt.% for other elements, respectively. Then, 50 μm Ti_6_Al_4_V powder was applied as the raw material. With a 30 μm laser spot, samples were fabricated layer-by-layer (50 μm) according to the CAD data. A laser power of 200 W and a velocity of 150 mm/s were applied. The process was strictly processed in an argon environment.

Scanning electron microscopy (SEM) was applied for the observation of the surface morphology of macroporous titanium alloy scaffolds. For the examination of biomechanical properties, compressive tests were conducted using a universal testing machine (880, MTS, USA). The maximum load capacity and loading rate were 500 kN load cell and 0.5 mm/min, respectively.

HA is the simplest glycosaminoglycan and a major constituent of the extracellular matrix. HA could provide compression strength, lubrication, and hydration within the ECM. The commercial hydrogel we applied in this study was mainly composed of HA, which could achieve sustained release and augment the retention and stability of sEVs (HyStem™ C, Merck). Then, 1.5 ml of sEV and sEV-20a solution (50 wt%) were mixed with HA solution and injected into the pores of porous titanium. The mixed solution turned into a gel and was observed by SEM for morphology.

For analysis of sustained release, a CD63 ELISA kit (System Biosciences) was applied following the instructions provided. In short, 50 μl of prepared protein standards and sEVs were added to the microtiter plate and incubated at 37 °C for 1 h. Then, approximately 50 μl of the CD63 primary antibody was added to each well and cultured for 1 h. Next, 50 μl of the secondary antibody was added to each well, which was cultured for 1 h at room temperature. Approximately 50 μl of substrate was added and cultured for 5–15 min. Finally, the quantitative results were acquired by measuring absorbance via a spectrophotometric plate reader at a wavelength of 450 nm.

### In vivo responses to the H-sEV-20a coating porous titanium alloy scaffold

#### The establishment of the osteoporotic rat model

We were authorized to carry out animal surgical operations by the Animal Research Committee of Shanghai Sixth People’s Hospital. For the surgical operations, we used thirty-six 12-week-old female Sprague-Dawley (SD) rats (approximately 150–200 g) in this research. For the verification of osteoporosis and the in vivo bone defect model, the rats were divided into two groups: the control group (*n* = 6) and the OVX group (*n* = 30). No ovariectomy was performed in the control group. The other thirty rats underwent ovariectomy and were used for the following examinations and operations. Afterwards, we verified whether the rat model of osteoporosis was successfully established through micro-CT (Skyscan 1176, Kontich, Belgium). BV/TV and BMDs were used as indicators.

#### Animal operation

After anesthetization with 3% phenobarbital sodium intraperitoneally (0.8 ml/kg), an osteoporotic rat model that we established for 2 months was used for the operation. We exposed the femur condyles and drill a 3.5 mm-diameter and 5 mm-depth round bone defect. We divided randomly four groups as follows: porous titanium alloy (Ti), porous titanium alloy+hydrogel (Ti-H), Ti-H + sEV (Ti-H-sEV), and Ti-H-sEV + 20a (Ti-H-sEV-20a) (*n* = 6). The tissues were carefully handled after the operation. After the operation, the rats were given sufficient food and water. Antibiotics were applied intramuscularly to each rat after surgery for 3 days. Three months later, the rats were euthanized, and femurs were obtained. Then, 4% paraformaldehyde was used to fix the acquired femurs. Then, micro-CT, fluorochrome labeling analysis, and van Gieson staining were conducted.

#### Micro-CT

Micro-CT (Skyscan 1176, Kontich, Belgium) was applied for the determination of new bone formation. After the specimens were harvested 12 weeks after the operation, the undecalcified femurs were scanned at 18 μm resolution. Sagital and coronal images were acquired by CTVox. Then, BV/TV and BMDs data were obtained by using image analysis software.

#### Sequential fluorescent labeling

The newly formed bone was assessed by trichromatic sequential fluorescent labeling. In short, 25 mg/kg (5 mg/ml) tetracycline (TE, Sigma), 30 mg/kg (6 mg/ml) alizarin red (AL, Sigma), and 20 mg/kg (4 mg/ml) calcein (CA, Sigma) were administered through intraperitoneal injection at 3, 6, and 9 weeks after surgery.

#### Histological analysis

Gradient concentrations of alcohols (50%, 70%, 75%, 80%, 85%, 90%, 95%, and 100%) and xylene were applied for the dehydration and transparency of the samples. After that, the femurs were treated with solution A composed of 80 ml of methyl methacrylate (MMA) and 20 ml of dibutyl phthalate (DBP) for 2 days and solution B composed of 80 ml of MMA, 20 ml of DBP, and 5 g benzoyl peroxide for 2 days. Subsequently, all femurs were placed in glass bottles filled with solution B for polymerization. Subsequently, for further observation, 150-μm-thick undecalcified bone slicing was performed via a microtome (Leica). Then, we used universal glue to fix the sections on the surface of a glass slide. The observation of the fluorescent labeling of the undecalcified bone slicing was carried out by operating a confocal laser scanning microscope (Leica). We set the excitation/emission wavelengths of 405/560–590 nm (tetracycline), 543/580–670 nm (alizarin red), and 488/500–550 nm (calcein). Then, after the samples were polished to ~ 50 μm, van Gieson’s picrofuchsin was implemented to assess the undecalcified bone sections of new bone formation. The newly formed bone was indicated by red. Six randomly selected sections were chosen to quantify new bone formation by ImageJ.

### The selection of the target of miR-20a

We predicted the target genes of miR-20a by three different databases, namely Starbase, miRDB, and miRWalk. Then, we used another anti-osteogenic database as well as the literature to screen the targets of miR-20a. We utilized Venn diagram acquiring the common genes among the potential target genes. The antibody for BAMBI was obtained from Abcam (ab203070).

### Luciferase report assay

We constructed luciferase reporter plasmids containing the 3′-UTRs of BAMBI as well as their corresponding mutated 3′-UTRs. In detail, the pMIR-REPORT Luciferase reporter vector (Ambion, USA) was inserted with the 3′-UTRs, and mutated 3′-UTR of BAMBI. Then, 293 T cells were seeded into 96-well plates and used for luciferase report assay. Lipofectamine 3000 (Invitrogen) and reporter plasmid as well as miR-20a mimics were co-transfected into each well 24 h later. Then, the Dual-Luciferase Reporter Assay System (E1910, Promega) was applied to measure Renilla and firefly luciferase activities.

### Statistics

**Fig. 1:**
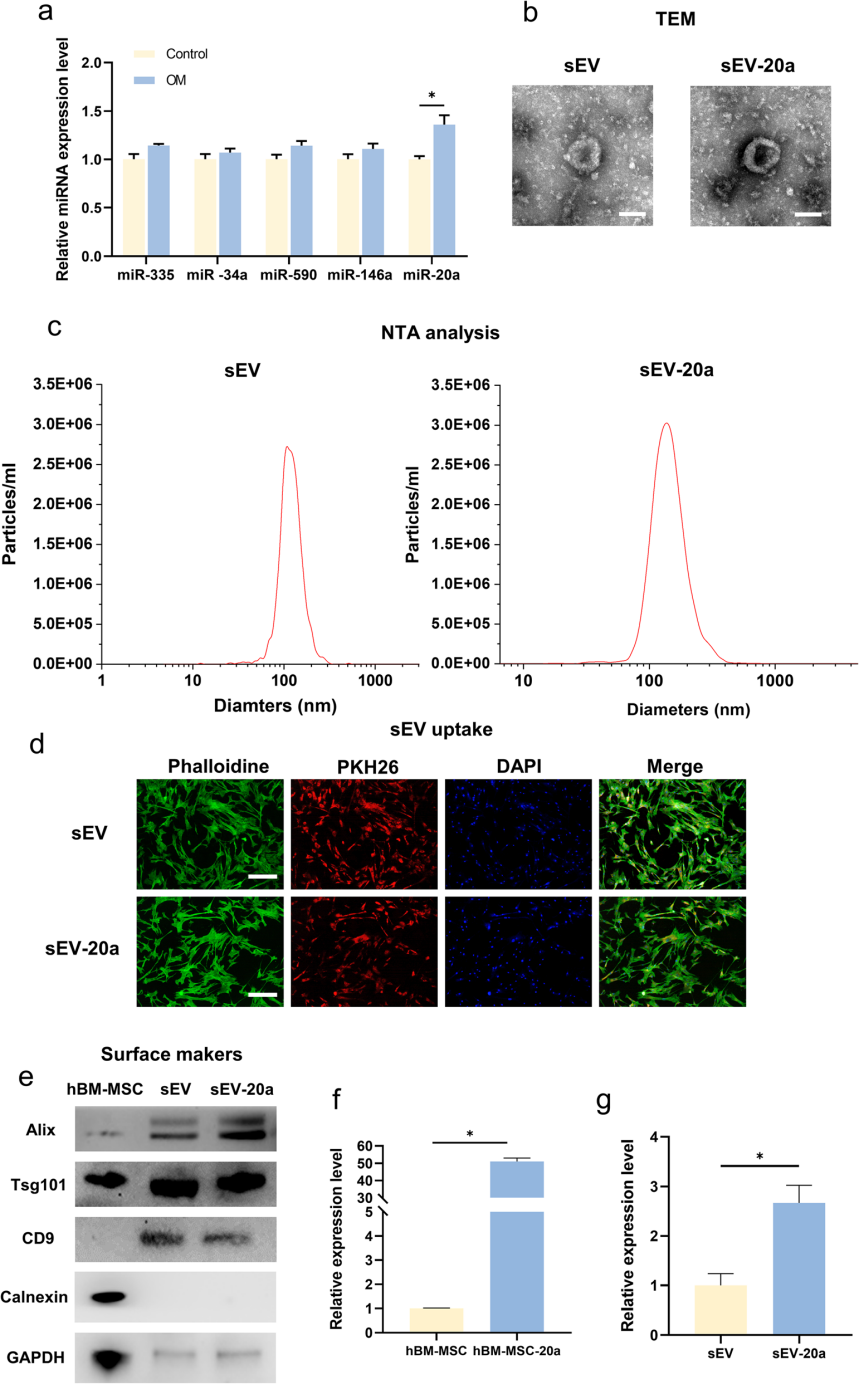
Selection of miR-20a and characterization of hBM-MSC-derived sEVs. **a** The selection of miR-20a among the five candidates after treated with OM. **b** The morphology of sEV and sEV-20a was analyzed by TEM. Scale bar = 100 nm. **c** The diameter of sEV and sEV-20a was measured by NTA. **d** The uptake of sEV by hBM-MSCs detected by fluorescence staining. Green, red and blue represent the cytoskeleton, sEV and nucleus respectively. Scale bar = 100 μm. **e** Western-blot was applied for the detection of the specific surface markers (Alix, Tsg101, CD9) of sEV and sEV-20a. Calnexin was used as a negative marker and GAPDH was applied for the internal reference. hBM-MSC group was used for control. **f** The relative expression of miR-20a in hBM-MSC overexpressing miR-20a. hBM-MSC group was used for control. **g** The relative gene expression of miR-20a in hBM-MSC-derived sEVs overexpressing miR-20a. sEV group was used for control (*p < 0.05)

**Fig. 2:**
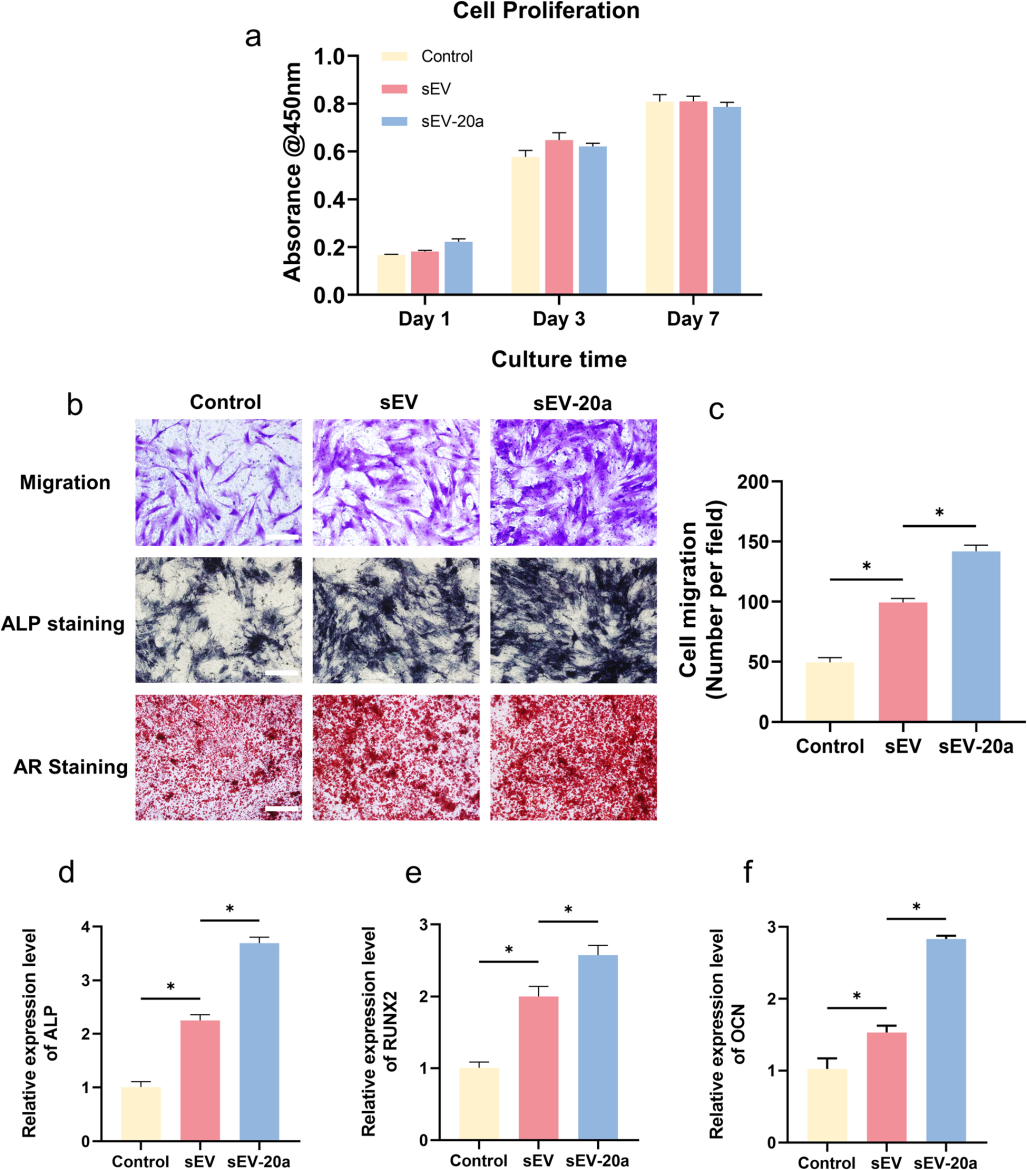
sEV-20a promotes the migration and osteogenic differentiation of hBM-MSCs in vitro. **a** Proliferation of hBM-MSCs cultured with sEV-20a by CCK-8 assay. **b**-**c** Cell migration of hBM-MSCs cultured with sEV-20a by transwell assay. Scale bar = 100 μm.ALP staining and ARS were applied for the assessment of osteogenic differentiation of hBM-MSCs by different treatments. Scale bar = 200 μm. **d**-**f** Relative gene expressions for ALP, RUNX2, OCN of hBM-MSCs by different treatments. GAPDH was used for normalization (*p < 0.05)

**Fig. 3:**
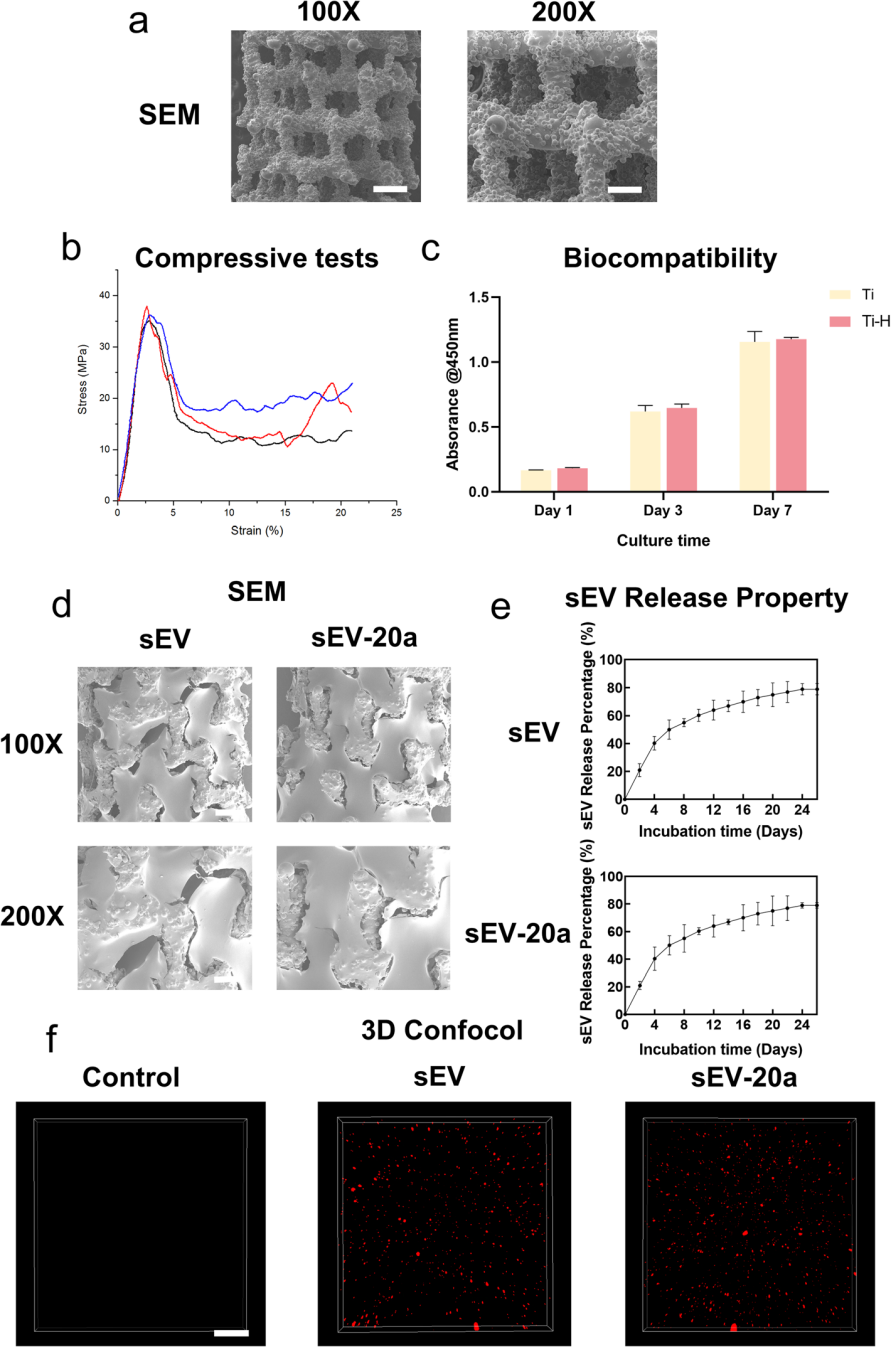
Characterization of porous titanium alloy. **a** The surface morphology of macroporous titanium alloy scaffolds were observed by SEM. Scale bar = 500 (100X) and 200 μm (200X). **b** Compressive tests were conducted using a universal testing machine to examine the biomechanical property of titanium alloy. **c** The cytocompatibility of Ti and Ti-H were detected using hBM-MSC by CCK-8 test. **d** The morphology of porous titanium alloy coated by H-sEV-20a via SEM. Scale bar = 400 (100X) and 200 μm (200X). **e** The release kinetics was measured by CD63 ELISA kit for 14 days for evaluation of the sustained release of sEVs (n = 3, *p < 0.05). **f** The distribution of sEVs incorporated in HA hydrogel was evaluated by 3D laser confocal microscopy. Scale bar = 200 μm. (*p < 0.05)

**Fig. 4:**
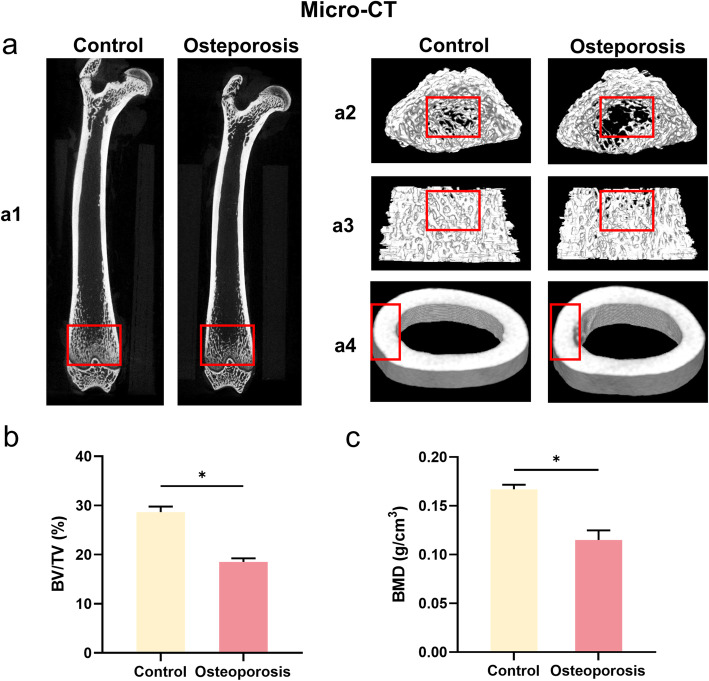
The OVX model verification of SD rats. **a** Micro-CT images showed the femur of OVX group had lower bone density compared with the Control group without OVX treatment. **b**-**c** Statistical analysis of BMD and BV/TV between OVX and the Control groups. (*p< 0.05)

**Fig. 5:**
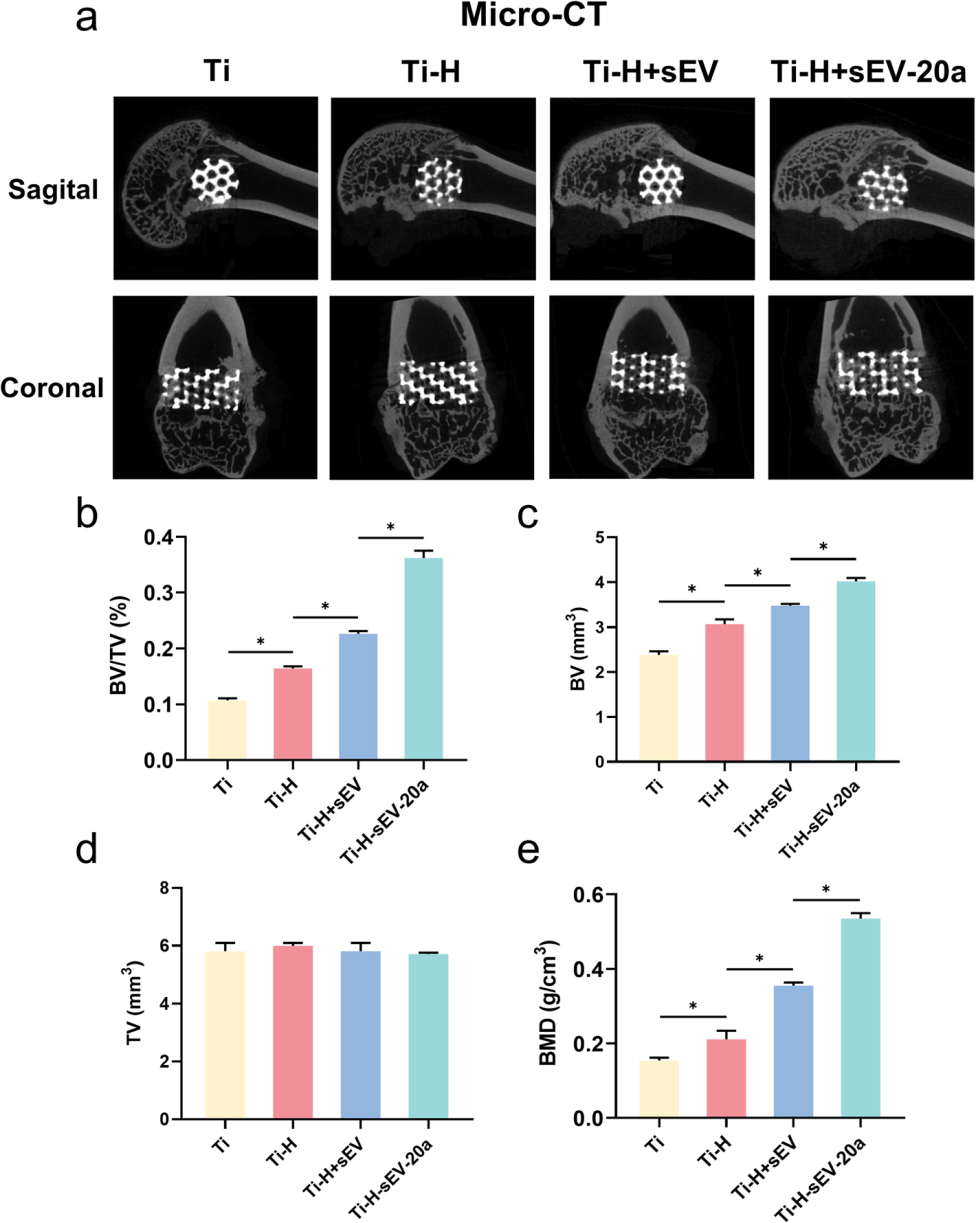
Ti-H-sEV-20a enhanced osteointegration evaluated by micro-CT in vivo. **a** Sagittal and coronal images of bone defects after 12 weeks after different treatments by micro-CT. **b**-**e** Different indicators including BV, TV, BV/TV and BMD were used for statistical analysis of micro-CT (*P < 0.05)

**Fig. 6:**
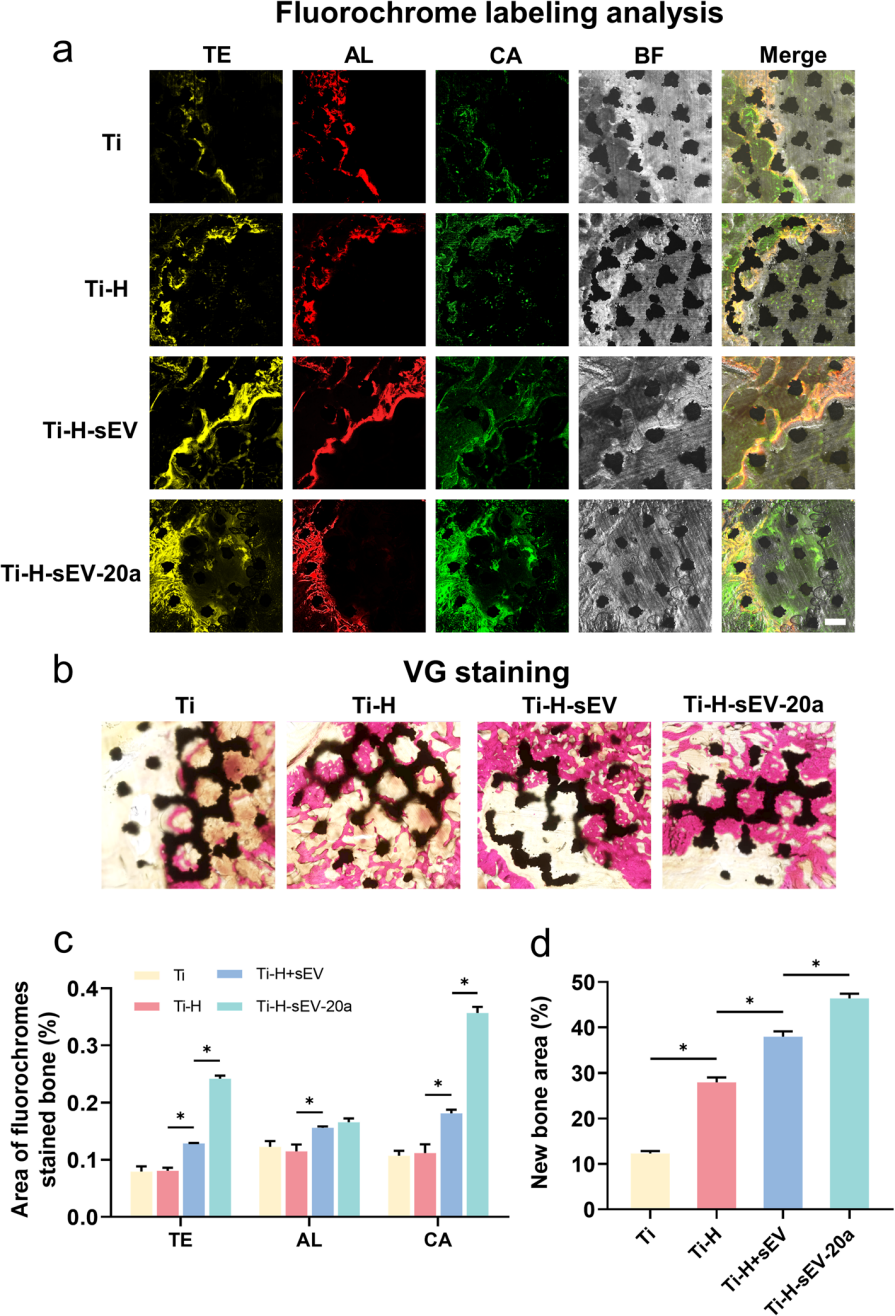
Ti-H-sEV-20a enhanced the osteointegration evaluated by histology in vivo. **a** The osteointegration results were shown by fluorochrome labeling analysis. The images showing yellow (TE), red (AL), green (CA) and bright filed (BF) were photographed by laser confocal microscopy. **b** The undecalcified specimens were stained with van Gieson’s picrofuchsin. Red area represented newly regenerated bone. Scale bar = 500 μm **c** TE, AL and CA staining area were assessed by statistical analysis. Scale bar = 500 μm. **d** The quantitative analysis of new bone formation by VG staining was calculated by Image J. (n = 6, * p< 0.05, the magnification was 40×)

**Fig. 7:**
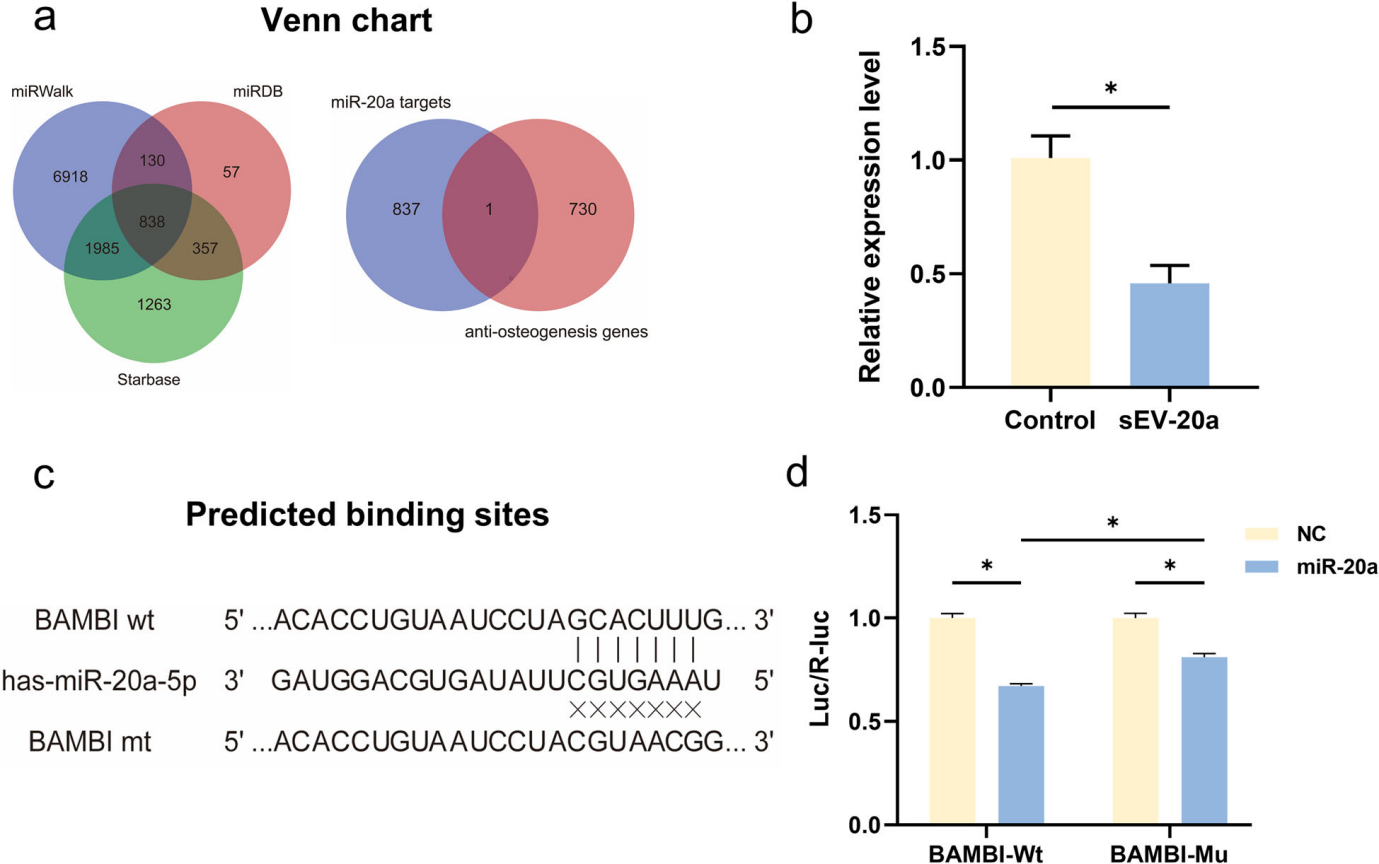
Bioinformatic analysis and verification of the targets of miR-20a. **a** Three online prediction tools such as miRWalk, Starbase and miRDB were applied for selecting the potential targets of miR-20a. Venn diagram was showed for screening of BAMBI. **b** The gene expression of BAMBI after treated with sEV-20a. **c** The predicted binding sites of miR-20a in the 3′-UTR of BAMBI-WT mRNA. **d** Luciferase report assay showed the luciferase activity of BAMBI-WT and BAMBI-Mu treated by miR-20a. Negative control (NC) was used as the control vector ( *p < 0.05)

**Fig. 8:**
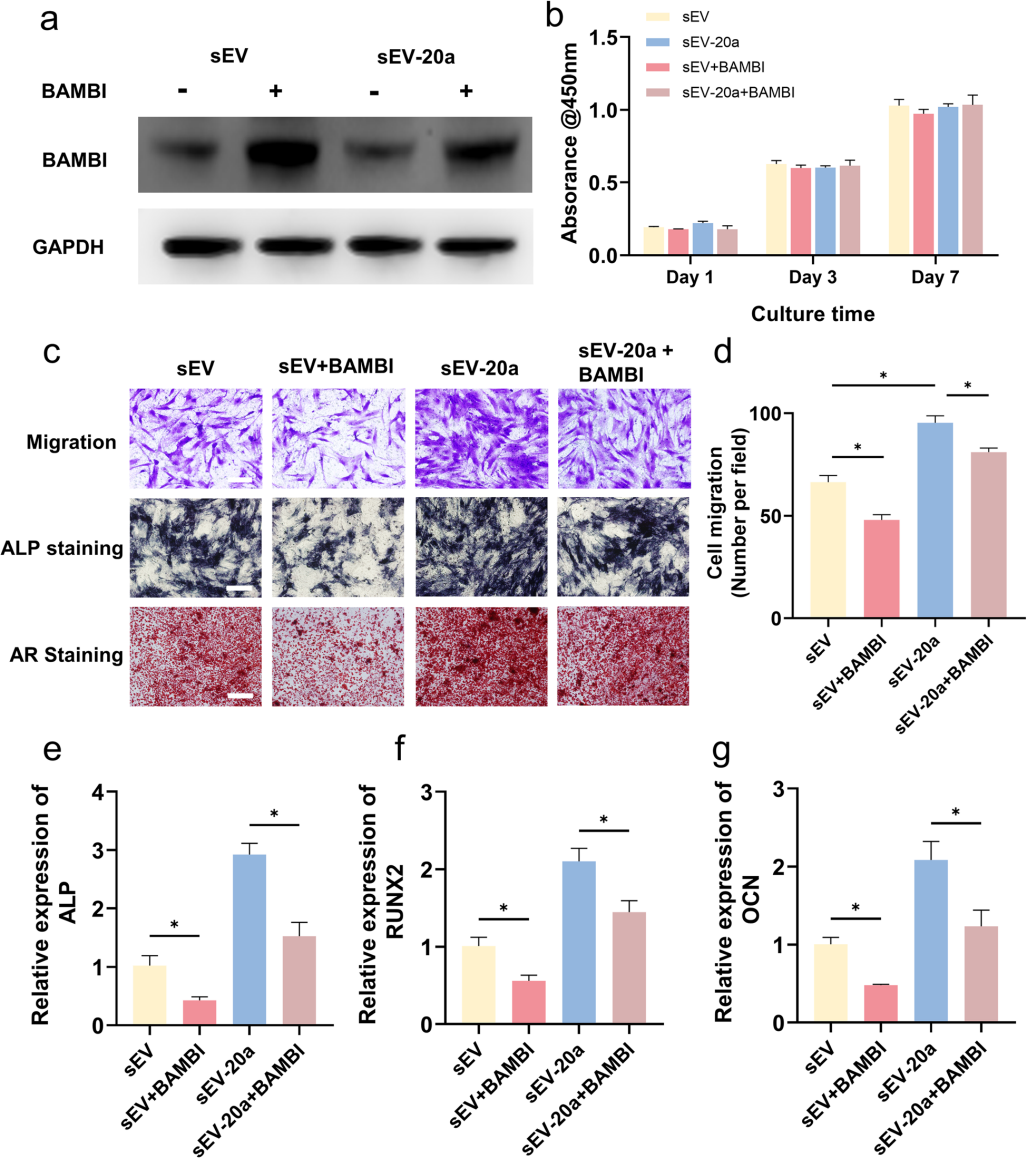
sEV-20a promotes the migration and osteogenic differentiation of hBM-MSCs through targeting BAMBI. **a** The protein expression level of BAMBI after the overexpression of BAMBI (+) and treated by sEV and sEV-20a by Western blot. **b** The proliferation of hBM-MSCs overexpressing BAMBI incubated with sEV and sEV-20a by CCK-8 assay. **c**-**d** Cell migration of hBM-MSCs overexpressing BAMBI cultured with sEV and sEV-20a by transwell assay. Scale bar = 100 μm. ALP staining and ARS for the assessment of osteogenic differentiation of hBM-MSCs overexpressing BAMBI cultured with sEV and sEV-20a. Scale bar = 200 μm. **e**-**g** Relative gene expression of ALP, RUNX2, OCN of hBM-MSCs overexpressing BAMBI treated with sEV and sEV-20a. GAPDH was used for normalization (n = 3, *p < 0.05)

**Fig. 9:**
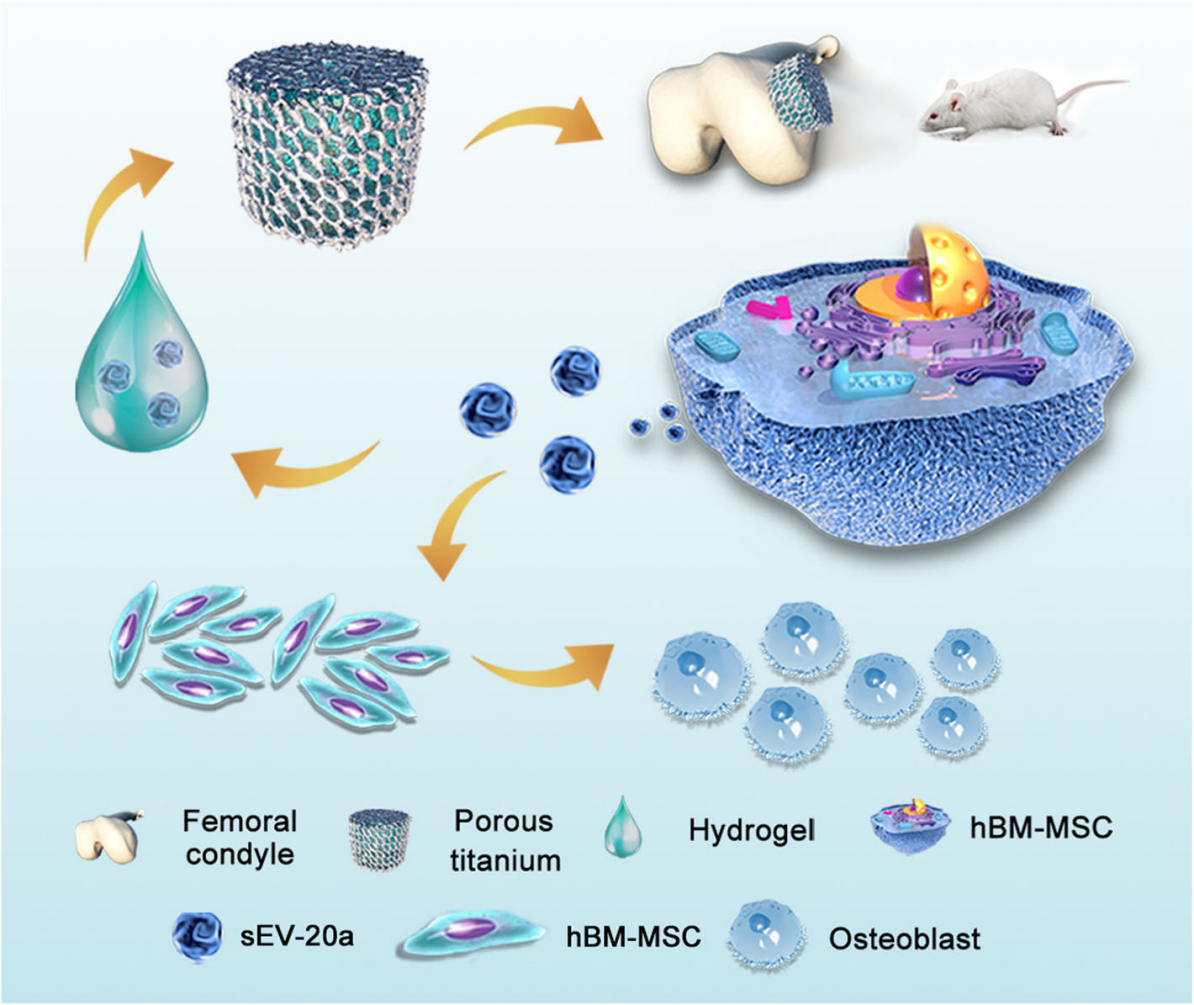
Schematic diagram depicts the detailed mechanisms of miR-20a on pro-osteogenesis and pro-osteointegration effects. In vitro, sEV-20a was successfully established and proved to promote the migration and osteogenesis of hBM-MSCs. In vivo, sEV-20a promoted osteointegration in an osteoporotic rat model. In addition, we proved that miR-20a could enhance osteogenesis by targeting BAMBI

Means ± standard error of the mean (SEM) was applied for the statistical analysis. The determination of significance was performed with Student’s t test (Fig. 1f, 4b, c, Fig. 7b), one-way ANOVA (Fig. 2c–f, Fig. 5b–e, Fig. 6d, Fig. 8d–g), and two-way ANOVA (Fig. 1a, Fig. 2a, Fig. 3c, Fig. 6c, Fig. 7d, Fig. 8b, Fig. 9). When the *P* value was < 0.05, the results were deemed statistically significant.

## Results

### The selection of miR-20a and the characterization of hBM-MSC-derived sEVs

To select the osteogenic miRNAs, we searched the literature and chose five miRNAs as potential candidates that positively regulate the osteogenic process: miR-335, miR-34a, miR-20a miR-590, and miR-146a. After we applied OM for 21 days, the expression of miR-20a was significantly upregulated in comparison with that of the control group, while the relative expression of other miRNAs remained the same (Fig. 1a). Thus, we identified miR-20a as a potential bioactive molecule for osteogenesis.

Then, we overexpressed miR-20a in hBM-MSCs and obtained the relevant sEVs overexpressing miR-20a. TEM results showed that the sEVs were round vesicles with main diameters of approximately 100–120 nm (Fig. 1b). The NTA results revealed the size distribution of the sEVs, and the sEV-20a and the sEVs displayed a single bell-shaped distribution with a peak diameter at approximately 120 nm and 130 nm, respectively (Fig. 1c). The uptake experiments demonstrated that the sEVs could be successfully endocytosed by hBM-MSCs and further exert their effects on recipient cells (Fig. 1d). There was no significant difference between the sEVs and sEV-20a in diameter and concentration. In addition, the relative protein expression of CD9, Tsg101, and Alix was positive, but the expression of calnexin was negative (Fig. 1e). Moreover, the qRT-PCR results demonstrated that hBM-MSCs and sEVs both overexpressed miR-20a, indicating the successful establishment of the engineered sEVs (Fig. 1f). The data proved the successful extraction and identification of sEVs as well as the overexpression of miR-20a in hBM-MSCs and hBM-MSC-derived sEVs.

### MiR-20a promoted the migration and osteogenesis of hBM-MSCs in vitro

CCK-8 tests were applied to study the proliferation of hBM-MSCs. Figure 2a shows that sEV-20a and sEV had no effect on the proliferation of hBM-MSCs in comparison with that of the other groups. Furthermore, migration is an important feature of cells, especially in tissue engineering, because this ability could help the surrounding cells move into the scaffolds and exert related biological functions. For the evaluation of migration, a Transwell assay was carried out and revealed that sEV-20a significantly promoted the migration of hBM-MSCs compared with the sEVs and Control (Fig. 2b, c). For the evaluation of the osteogenic differentiation ability, we performed ALP staining and ARS, showing that sEV-20a had the best osteogenic effect compared with the sEVs and control (Fig. 2b). Additionally, qRT-PCR was carried out, and we observed that osteogenesis-related gene expression of the ALP, RUNX2, and OCN genes was upregulated at day 14 in the sEV-20a group, which suggested the important role of miR-20a in osteogenesis (Fig. 2d–f).

To further verify our hypothesis, we used a miR-20a inhibitor (miR-20ai). Figure S1a illustrates that sEV-20a and sEV-20ai had no effect on the proliferation of hBM-MSCs compared with the cells in the other groups. Furthermore, sEV-20a significantly promoted the migration of hBM-MSCs, while sEV-20ai significantly inhibited the migration in comparison with the control group (Figure S1b-c). For the evaluation of, we performed ALP staining and ARS, showing that sEV-20ai significantly impaired osteogenesis in comparison with the control group (Figure S1b). Additionally, the osteogenesis-related gene expression of the ALP, RUNX2, and OCN genes was significantly upregulated at day 14 by sEV-20a and downregulated by sEV-20ai, showing the important role of miR-20a in osteogenesis (Figure S1d-f).

### Characterization of porous titanium alloy

The porosity of the macroporous titanium alloy scaffolds was approximately 75%, and the diameter of the pores was approximately 600 μm with pores filled with hydrogel (Fig. 3a, d). By a compression test, we found that macroporous titanium alloy scaffolds had a maximum compressive strength of 36.42 MPa, along with a Young’s modulus of 1.81 ± 0.28 GPa (Fig. 3b). The cytocompatibility was verified by CCK-8 assays, and the scaffold showed favorable properties for hBM-MSCs (Fig. 3c). Additionally, a CD63 sEV ELISA kit was applied to evaluate the sustained release of sEVs packed by hydrogel. At day 24, approximately 80% of the sEVs was released, and the amount ceased to increase in the following days (Fig. 3e). To validate the successful incorporation of sEVs into the hydrogel, we performed a 3D confocal laser scan and showed that the sEVs were labeled by the red fluorescent dye PKH26 and were distributed homogeneously (Fig. 3f). All these data indicated that the H-sEV-20a-coated macroporous titanium alloy scaffolds have favorable properties for bone weight bearing and are conducive to bone ingrowth.

### H-sEV-20a improved titanium alloy scaffold osteointegration in osteoporotic rats

#### Micro-CT analysis

For the osteointegration effect of miR-20a, we established an OVX-induced osteoporotic SD rat model, which was verified by micro-CT. The bone quality of the entire femur (a1), proximal femur (a2), middle femoral shaft (a3), and distal femur (a4) in the OVX group was worse than that in the Control group (Fig. 4a1-a4). Subsequently, micro-CT was applied for measuring BV/TV (%) and BMD (g/cm2) (Fig. 4b, c). The OVX group showed a significant decrease in BV/TV and BMD in comparison with the control group, further proving the successful establishment of the osteoporotic rat models.

To evaluate the pro-osteointegration effect of H-sEV-20a, we applied micro-CT analysis, which exhibited the morphology of the newly formed bone. More newly developed bone was observed in the Ti-H-sEV-20a group than in the other three groups (Fig. 5a). The quantification of the newly formed bone was conducted by CTVox, among which BV/TV was markedly higher in the Ti-H-sEV-20a group than in the Ti-H-sEV group, the Ti-H group, and the Ti group (Fig. 5b–d). The BMD, which reflects the minerals in the bone, was also significantly higher in the Ti-H-sEV-20a group than in the other three groups (Fig. 5e). From the above data, we can conclude that Ti-H-sEV-20a could remarkably enhance osteointegration.

#### Fluorochrome labeling analysis

After the micro-CT analysis, we prepared undecalcified bone slicing, which was observed by laser confocal microscopy to evaluate the early and late osteointegration between bone and the implant at weeks 3, 6, and 9 by TE, AL, and CA, respectively (Fig. 6a, c). At 3 weeks, the area of TE labeling (yellow) in the Ti-H-sEV-20a group was greater than that in the Ti-H-sEV group, the Ti-H group, and the Ti group. These results suggested that sEV-20a enhanced early osteointegration. At 6 weeks, the AL labeling area (red) in the Ti-H-sEV-20a group was higher than that in the Ti-H-sEV group, Ti-H group, and Ti group. At 9 week, the CA labeling area (green) in the Ti-H-sEV-20a group was significantly greater than that in the other groups. Thus, we further verified that miR-20a could promote late osteointegration as well.

#### Van Gieson staining

Van Gieson (VG) solution is composed of picric acid and acid fuchsin and is used for staining new bone formation. After stained by VG solution, the newly formed bone showed a red color (Fig. 6b). Furthermore, by ImageJ, we found that the Ti-H-sEV-20a group showed markedly more newly formed bone than the Ti-H-sEV, Ti-H, and Ti groups (Fig. 6d).

The abovementioned results of micro-CT analysis, fluorochrome labeling analysis, and van Gieson staining together verified that sEV-20a has a better pro-osteointegration effect in vivo than the other treatments*.*

### sEV-20a enhanced the migration and osteogenic differentiation of hBM-MSCs by targeting BAMBI

To further explore the detailed mechanism by which miR-20a promotes osteogenesis, we also screened the downstream target of miR-20a by bioinformatics methods as well as the literature and finally identified BAMBI as a potential target (Fig. 7a). qRT-PCR verified that the gene expression of BAMBI was significantly downregulated after being treated with miR-20a (Fig. 7b). A luciferase reporter assay demonstrated that miR-20a directly inhibits BAMBI-Wt. However, after mutation of the binding site in the 3′UTR of BAMBI (BAMBI-Mu), the regulation of BAMBI by miR-20a was significantly weakened, proving that BAMBI was a target of miR-20a (Fig. 7c, d).

Furthermore, to validate the regulatory effect of miR-20a on BAMBI, we overexpressed BAMBI in hBM-MSCs. Figure 8a shows that the protein expression of BAMBI was significantly downregulated after treated with sEVs and sEV-20a. Additionally, the migration and osteogenic effects of hBM-MSCs in the sEV-BAMBI and sEV-20a + BAMBI groups were significantly impaired after the overexpression of BAMBI compared with those of the sEV and sEV-20a groups, as shown in Fig. 8b–g, but the proliferative ability was not affected. In conclusion, all these data verified the pro-osteogenic effect of miR-20a on hBM-MSCs by targeting BAMBI in vitro.

## Discussion

The stability of implants after surgery is essential for successful osteointegration, which is defined as the bonding between the newly formed bone and surgical implants; thus, surgical implants have a tight connection with the surrounding bone tissue, avoiding the loosening of the implants [2]. However, implants applied in orthopedic surgery for osteoporotic patients could show implant loosening and instability because of decreased osteogenesis, resulting in poor osteointegration [2]. However, poor osteointegration in turn could also aggravate implant loosening, becoming a vicious cycle. It has been reported that 20 μm of oscillating motion of implants has little effect on osteointegration, while 40 and 150 μm of oscillating motion cannot effectively support bone ingrowth [20]. Therefore, it is imperative to break this vicious cycle by improving osteointegration and avoiding implant loosening.

Elderly patients suffer from osteoporosis due to variations in biological molecules such as estrogen, growth hormones, or parathyroid hormone, leading to poor osteogenesis. For osteogenesis, miRNAs have been extensively reported to play important roles in this process. For example, it has been shown that the osteogenesis of mesenchymal stem cells could be regulated by miR-16-2* by targeting WNT5A [20]. To identify miRNAs exerting a pro-osteogenic effect, we selected miR-20a, which is reported to have pro-osteogenic effects [22–25]. In addition, a previous study proved that upregulating the expression levels of miR-20a would enhance the differentiation of BM-MSCs [22]. The reason why miR-20a could enhance osteogenic differentiation may be regulation of CKIP-1 expression or the BMP signaling pathway and mineralization by targeting the BMP-2 transcript [24–26]. Our in vitro experiments provided additional evidence that miR-20a was significantly upregulated after treatment with osteogenic induction medium.

MSCs are characterized by self-renewal and multidifferentiation, which lay the foundation of many promising therapies. Many researchers believe that MSCs have an important role due to paracrine mechanisms such as sEVs and growth factors [27–29]. Since MSC transplantation therapies have some potential risks, such as ethical issues and immune rejection, MSC-derived sEVs were selected to replace MSCs to reduce the MSC-related risks [30]. As a kind of nanocarrier, stem cell-derived sEVs have been applied for bone research. For example, hBM-MSC-sEVs restored the osteogenic ability of osteoblasts and increased osteogenic gene expression, thus improving osteoporosis [31]. Moreover, sEVs derived from human adipose mesenchymal stem cells (hASCs) overexpressing miR-375 were incorporated into hydrogels and promoted calvarial bone regeneration by targeting IGFBP3 [32]. Additionally, dimethyloxaloylglycine-stimulated sEVs derived from hBM-MSCs were shown to enhance bone regeneration by proangiogenic effects [12]. As a potential tool, sEVs could prevent miR-20a from being degraded by enzymes. In addition, sEVs could be endocytosed by recipient cells, further exerting their biological influence and avoiding the shortcomings of cell therapy, such as potential immunological rejection and risk of aneuploidy [33–35]. In this study, we verified that gene expression was significantly upregulated in both sEV-20a and recipient hBM-MSCs, which demonstrated that sEVs were endocytosed and effectively released.

Furthermore, BAMBI has a structure that resembles the TGF-β type I receptor but lacks an intracellular serine/threonine kinase domain. Additionally, BAMBI could inhibit osteogenic differentiation of hBM-MSCs [25]. A previous study demonstrated that miR-20a could reverse the osteogenic differentiation of hBM-MSCs by targeting BAMBI and coregulating the bone morphogenetic protein (BMP)/RUNX2 signaling pathway, a key pathway related to osteogenesis [25]. Therefore, BAMBI reacts with BMP ligands and has the ability to act as a pseudo receptor for antagonizing the TGF-β/BMP signaling pathway by suppressing the formation of active ligand-receptor complexes, thus indirectly inhibiting the effect of BMP ligands [13]. The gene and protein expression of BAMBI was significantly downregulated, while the related osteogenic genes ALP, RUNX2, and OCN were significantly upregulated by miR-20a intervention. Furthermore, we observed that the overexpression of BAMBI inhibited the migration and differentiation of hBM-MSCs, and miR-20a could counteract this effect by directly binding the 3′UTR of BAMBI.

Porous titanium alloys have many merits, such as pore size and porosity. Porous titanium with a pore size of ~ 600 μm, which is beneficial for cell growth and the transport of nutrients, biomolecules, and metabolic waste, is an ideal for the migration and differentiation of BM-MSCs. Enhanced new bone formation and the formation of blood vessels are observed when the pore size is greater than 300 μm [36]. Additionally, scaffolds with pore sizes of 600 μm and 900 μm were proven to have better ingrowth capability than scaffolds with 300 μm pore sizes [37]. Therefore, our 3D porous titanium alloy with a pore size of ~ 600 μm is highly applicable for osteointegration. In addition, a 3D porous structure with a porosity of 75% lays a foundation for the incorporation of bioactive molecules that could synergistically exert pro-osteointegration effects. Nevertheless, the large pore size of porous titanium alloy is also a drawback, which will result in the burst release of sEVs with a diameter of 30–150 nm. Therefore, we chose HA hydrogel, a traditional biocompatible scaffold with a favorable 3D network for the sustained delivery of sEVs [38, 39]. The release curve in this study verified that the amount of sEV HA hydrogel released was time-dependent, increasing to approximately 80% after 5 weeks. During this period, the sEVs were released, and the BM-MSCs around the bone defect migrated to the porous titanium alloy to proliferate, differentiate, and deposit the extracellular matrix, thus promoting osteogenesis. The in vivo results demonstrated that the Ti-H composite had a better effect than titanium alone and that Ti-H-sEV-20a treatment had the best pro-osteointegration and synergetic effect.

## Conclusion

To conclude, we explored the role of sEV-20a in promoting porous titanium alloy osteointegration by targeting BAMBI via a pro-osteogenic effect. This kind of stem cell therapy will provide appealing candidates for solving the issue of implant loosening resulting from osteoporosis.

## Supplementary Information

**Additional file 1.**

## Data Availability

The data supporting our findings can be acquired by the corresponding author Lili Yang.

## Abbreviations

hBM-MSC: Human bone marrow mesenchyme stem cell
ALP: Alkaline phosphatase
ARS: Alizard red staining
MSC: Mesenchyme stem cell
Small EV: sEV
sEV-20a: sEV overexpressing miR-20a
SEM: Scan electron microscopy
TEM: Transmission electron microscope
NTA: Nanoparticle tracking analysis
RUNX2: Runt-related transcription factor 2
OCN: Osteocalcin
BV/TV: Bone volume to total bone volume
BMDs: Bone mineral densities
