# Supplementary material for: MSC-derived small extracellular vesicles overexpressing miR-20a promoted the osteointegration of porous titanium alloy by enhancing osteogenesis via targeting BAMBI
Source: Stem Cell Res Ther. 2021 Jun 16;12:348. doi: 10.1186/s13287-021-02303-y (PMC8207591; doi:10.1186/s13287-021-02303-y)
Supplement: Supplementary file 1 — Additional file 1. [file 13287_2021_2303_MOESM1_ESM.docx]

**Figure S1.** MiR-20ai inhibited the migration and osteogenic differentiation of hBM-MSCs *in vitro*. (A) Proliferation of hBM-MSCs cultured with sEV-20a and sEV-20ai by CCK-8 assay. (B-C) Cell migration of hBM-MSCs cultured with sEV-20a and sEV-20ai by transwell assay. Scale bar=100μm.ALP staining and ARS were applied for the assessment of osteogenic differentiation of hBM-MSCs by different treatments. Scale bar=200μm. (D-F) Relative gene expressions for *ALP, RUNX2, OCN* of hBM-MSCs by different treatments. *GAPDH* was used for normalization (n=3, *p<0.05).

**Table. S2 Primer sequences used in qRT-PCR**

| Gene | Primers (F=forwards; R=reverse) |
| --- | --- |
| *RUNX2* | F: 5' TGGTTACTGTCATGGCGGGTA 3' |
|  | R: 5' TCTCAGATCGTTGAACCTTGCTA 3' |
| *OCN* | F: 5' CACTCCTCGCCCTATTGGC 3' |
|  | R: 5' CCCTCCTGCTTGGACACAAAG 3' |
| *ALP* | F: 5' ACTGGTACTCAGACAACGAGAT 3' |
|  | R: 5' ACGTCAATGTCCCTGATGTTATG 3' |
| *BAMBI* | F: 5' ATGCTCTCCCGTTTGCACTAC 3' |
|  | R: 5' AGGATCTTATCGTTGCTGAGGT 3' |
| *RNU6* | F: 5' GCT TCG GCA GCA CAT AT 3' |
| GAPDH  *18S* | R: 5' ATT TGC GTG TCA TCC TTG 3'  F: 5'- CGACCACTTTGTCAAGCTCA -3'  R: 5'- AGGGGTCTACATGGCAACTG -3'  F: 5'-GGACAGGATTGACAGATTGATAG-3'  R: 5'-CTCGTTCGTTTATCGGAATTAAC-3' |
